# Supplementary material for: Synthesis of Novel Pyrazole Derivatives Containing Phenylpyridine Moieties with Herbicidal Activity
Source: Molecules. 2022 Sep 23;27(19):6274. doi: 10.3390/molecules27196274 (PMC9571195; doi:10.3390/molecules27196274)
Supplement: Supplementary file 1 [file molecules-27-06274-s001.zip › molecules-1903290-Supplementary.pdf]

molecules

Supporting Information

Synthesis of Novel Pyrazole Derivatives Containing Phenylpyridine Moieties with Herbicidal Activity

Zengfei Cai [1], Wenliang Zhang [1], Zhongjie Yan [2] and Xiaohua Du [1,*]

[1] Catalytic Hydrogenation Research Center, Zhejiang Key Laboratory of Green Pesticides and Cleaner Production Technology, Zhejiang Green Pesticide Collaborative Innovation Center, Zhejiang University of Technology, Hangzhou 310014, China
[2] Agrowin (Ningbo) bioscience Co., Ltd., Ningbo, 315100, China
* Correspondence: duxiaohua@zjut.edu.cn

Contents

^{1}H NMR, ^{13}C NMR and HRMS spectra of target compounds 6a-6e and **7a–7e**..2

1. ^{1}H NMR, ^{13}C NMR and HRMS spectra of target compounds 6a–6e and 7a–7e

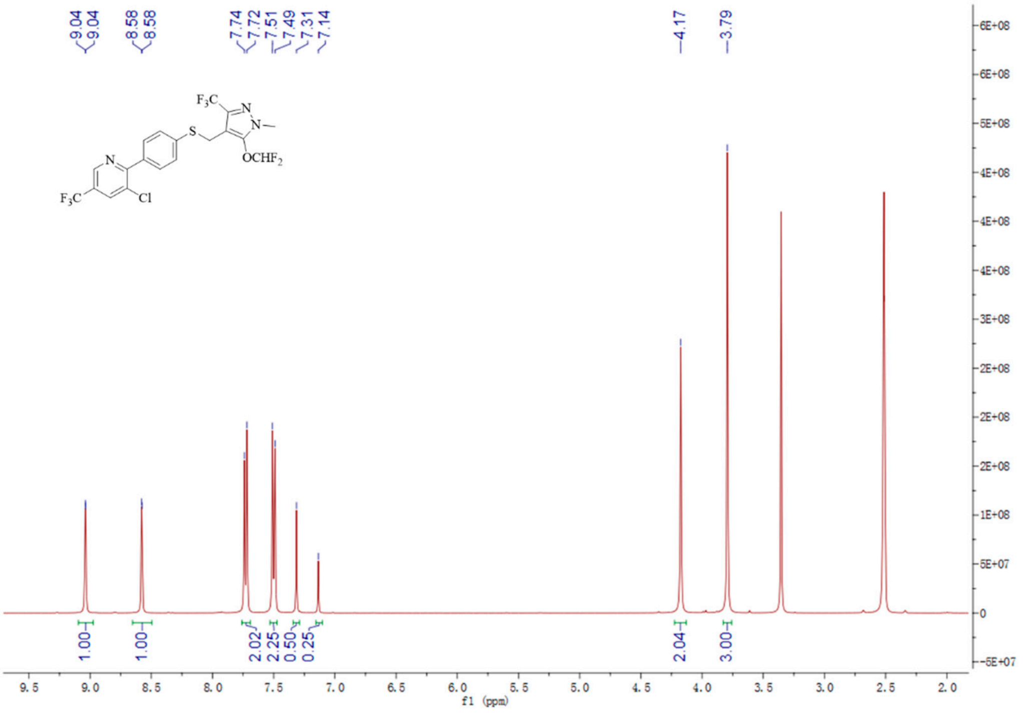

Figure S1. ^{1}H NMR of compound **6a.**

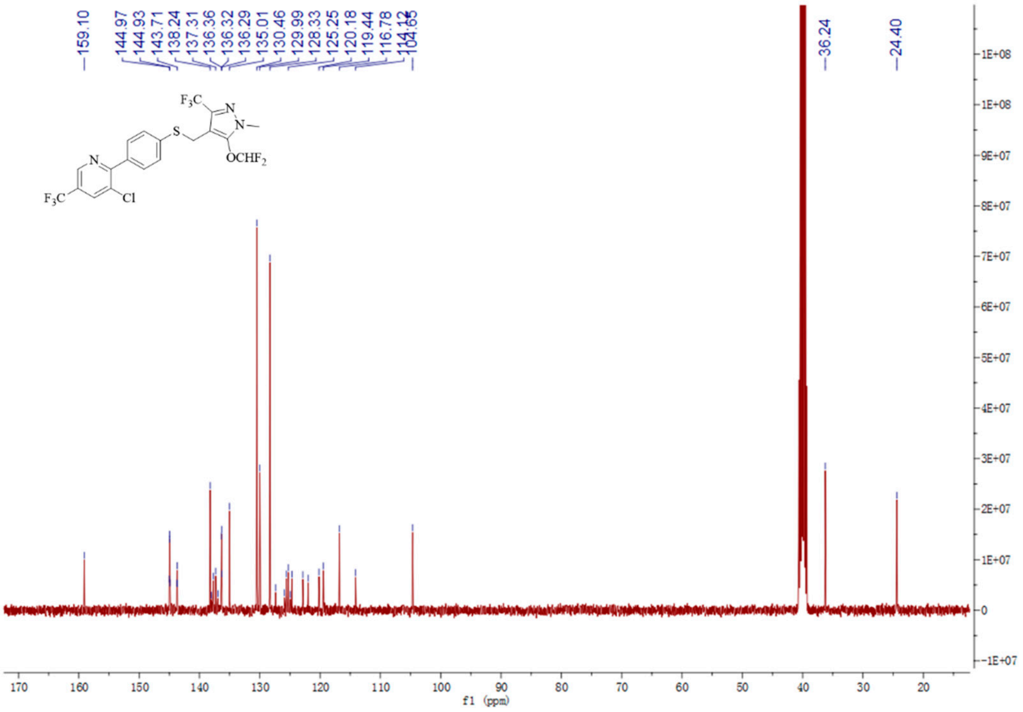

Figure S2. ^{13}C NMR of compound **6a.**

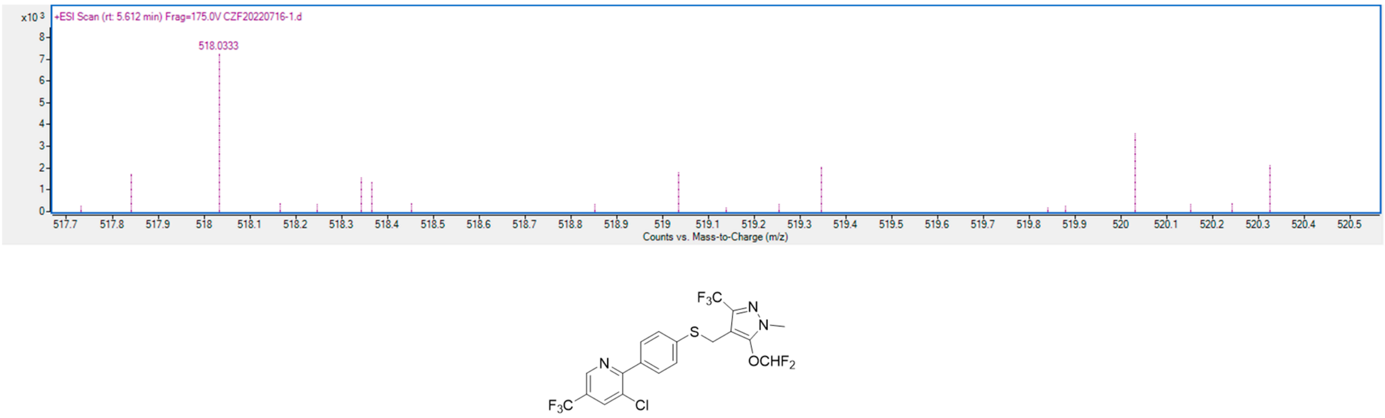

Figure S3. HRMS of compound **6a.**

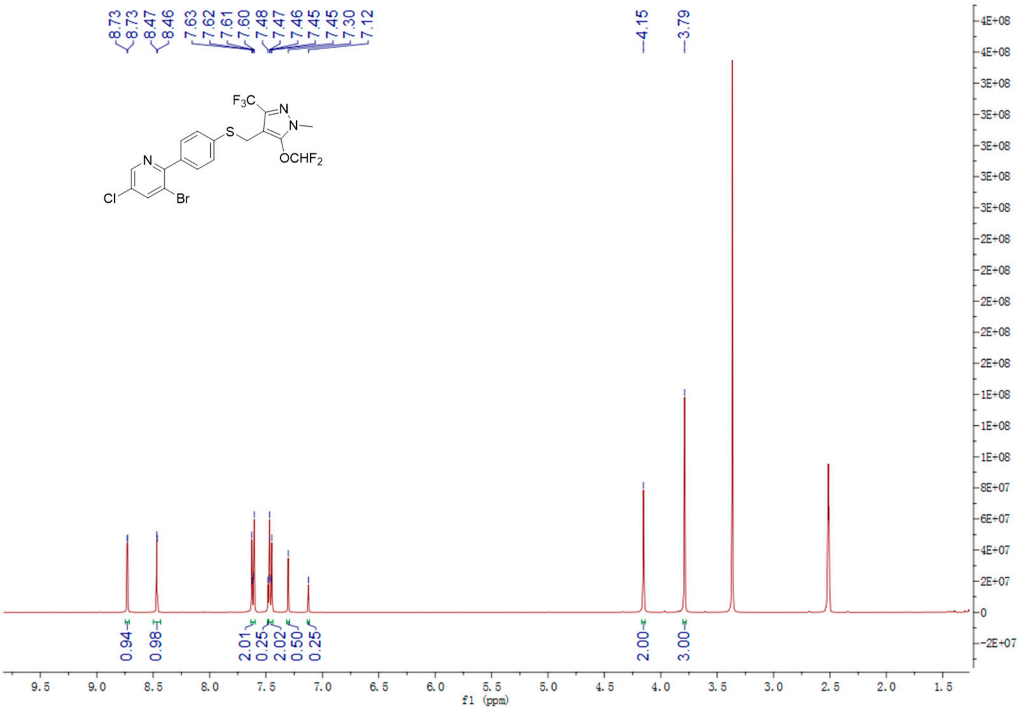

Figure S4. ^{1}H NMR of compound **6b.**

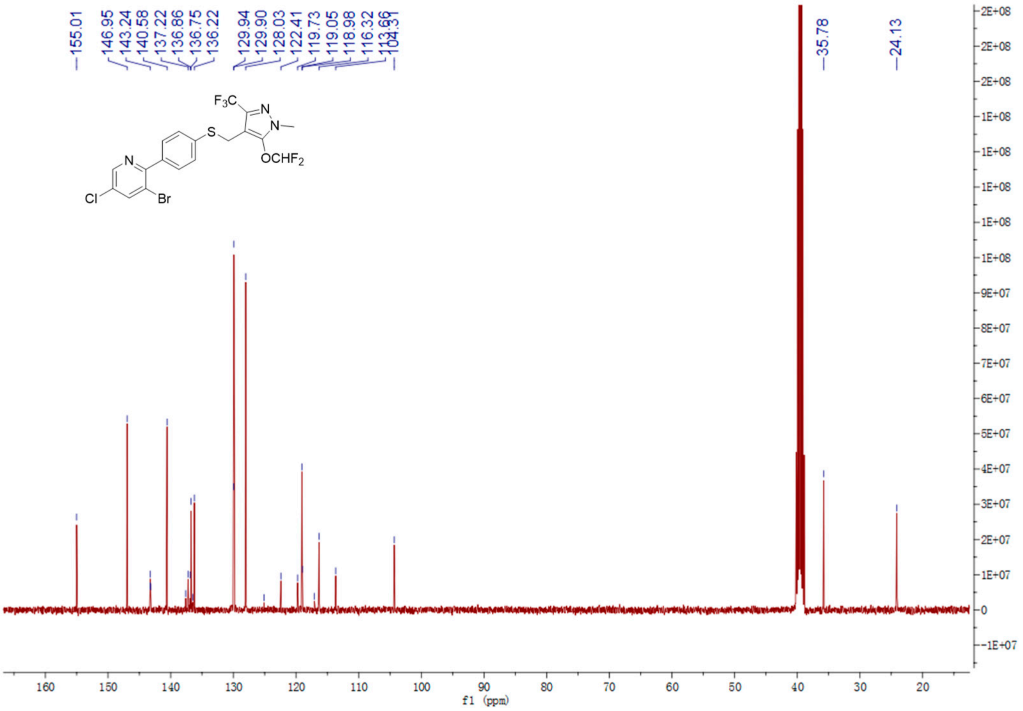

Figure S5. ^{13}C NMR of compound **6b.**

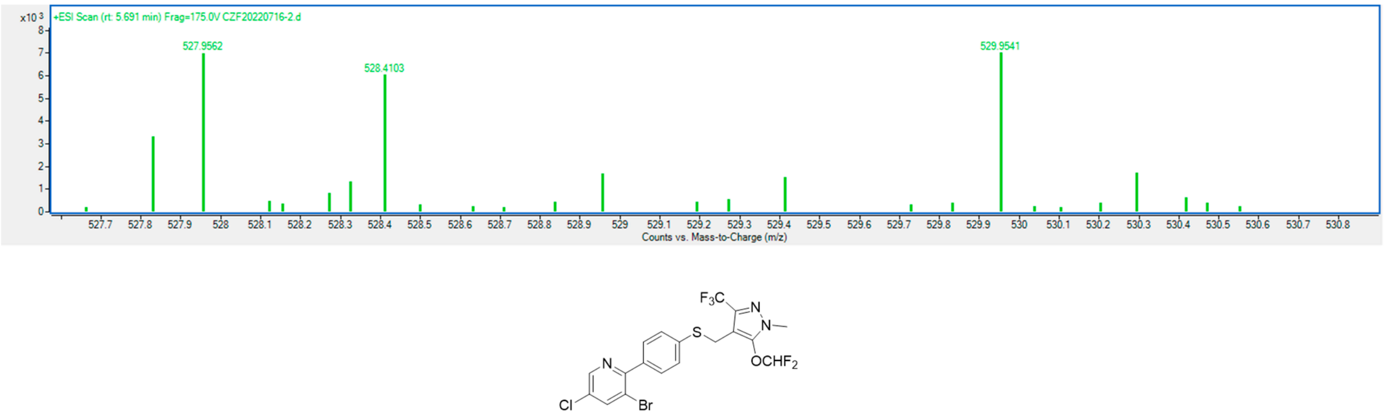

Figure S6. HRMS of compound **6b.**

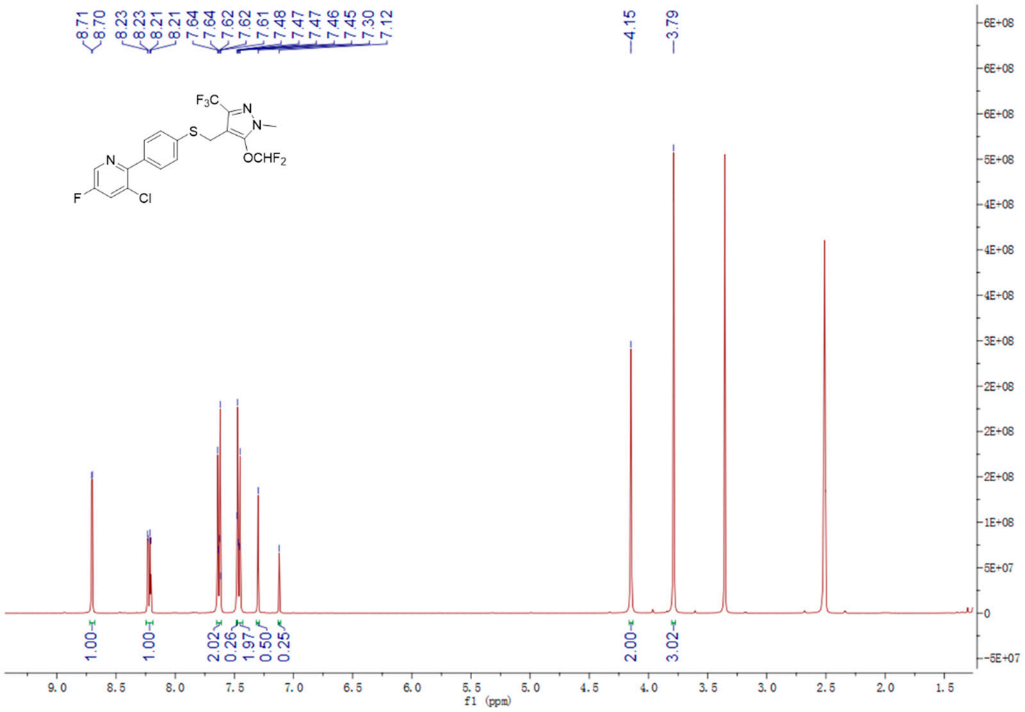

Figure S7. ^{1}H NMR of compound **6c.**

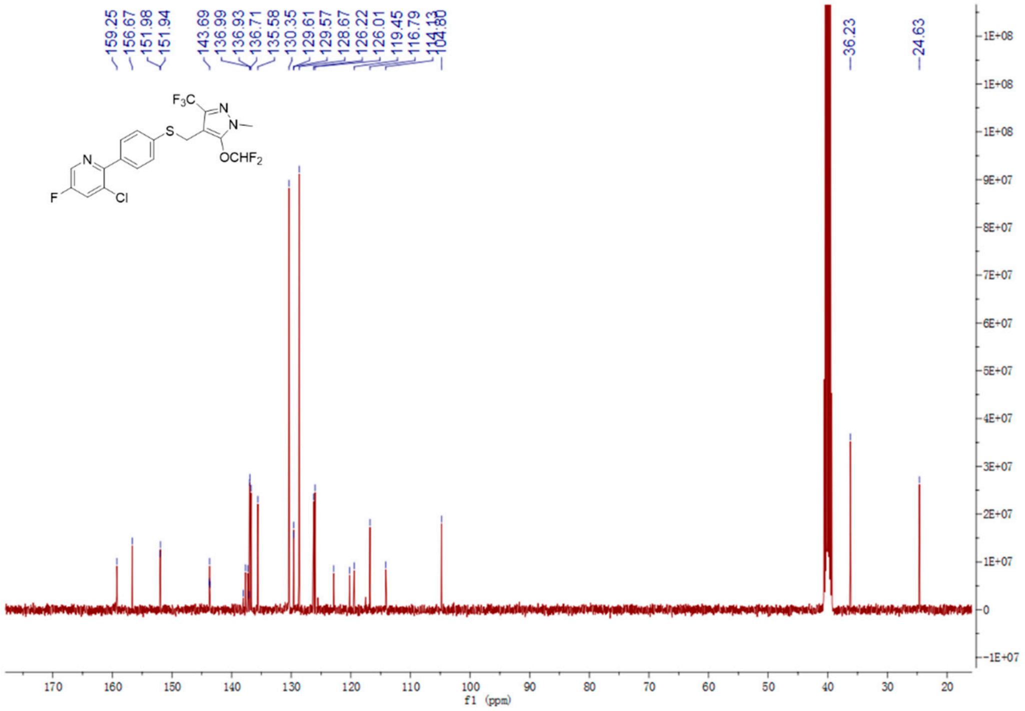

Figure S8. ^{13}C NMR of compound **6c.**

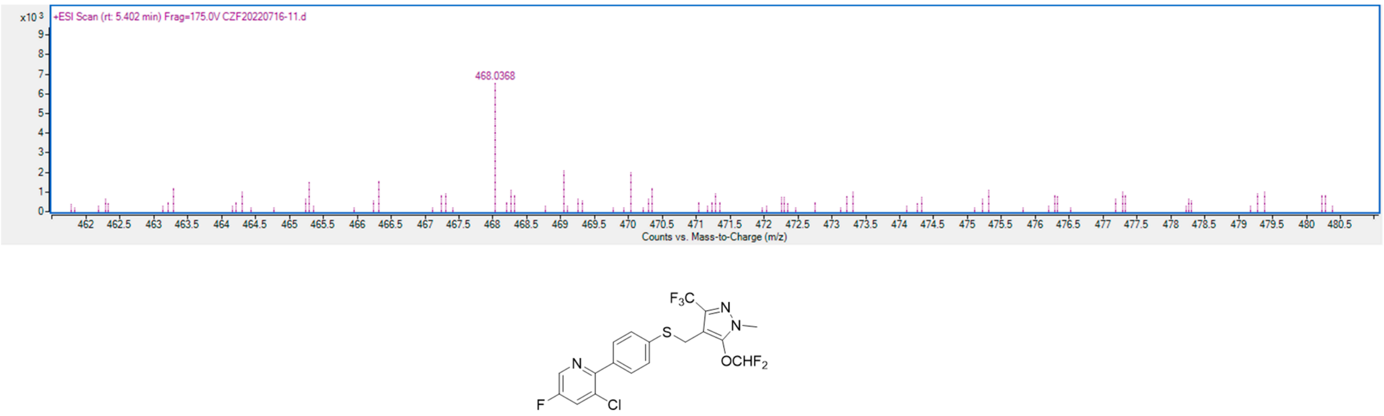

Figure S9. HRMS of compound **6c.**

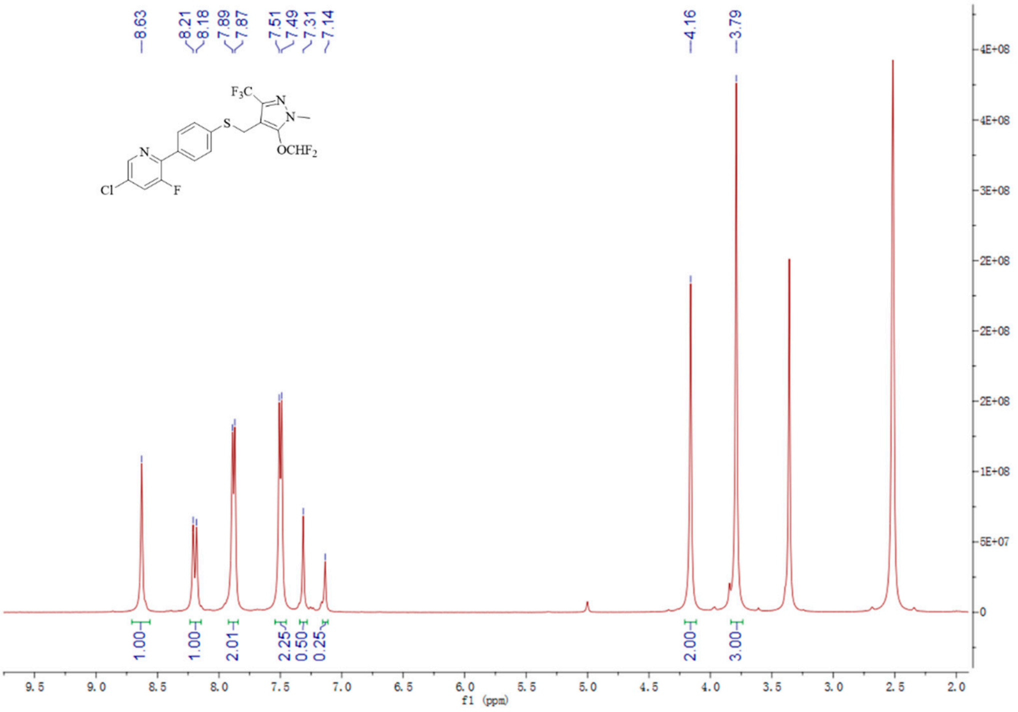

Figure S10. ¹H NMR of compound **6d.**

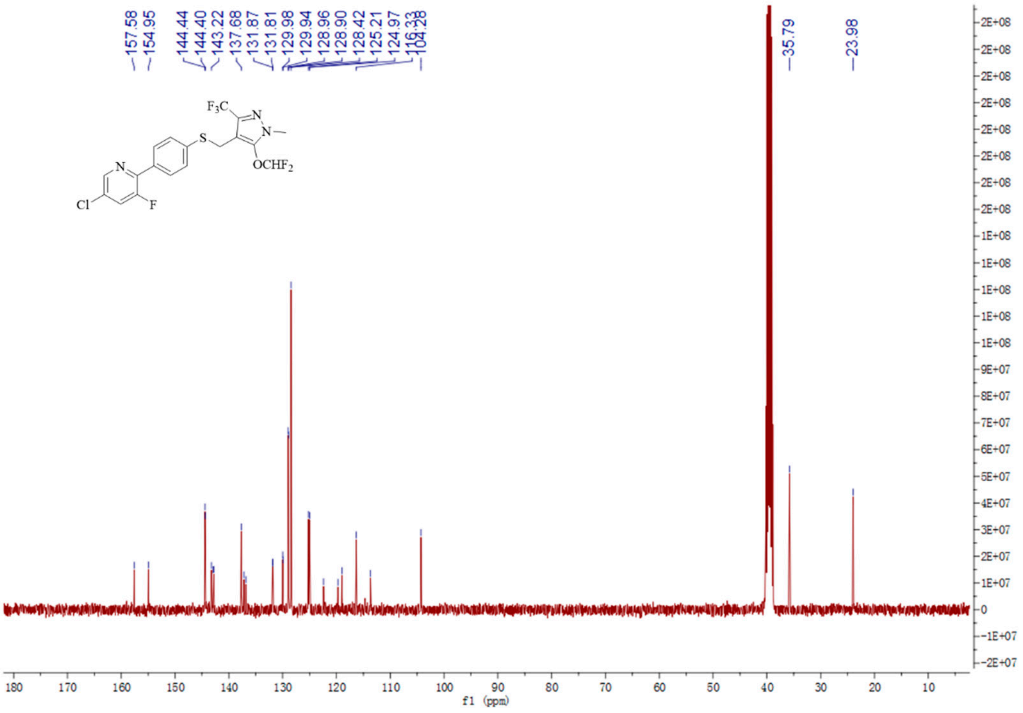

Figure S11. ^{13}C NMR of compound **6d.**

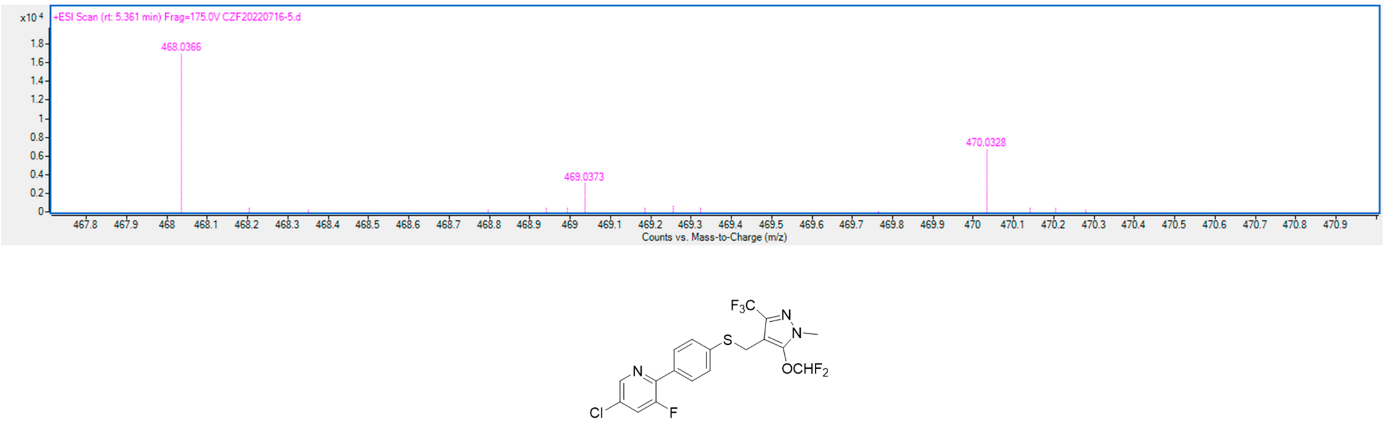

Figure S12. HRMS of compound **6d.**

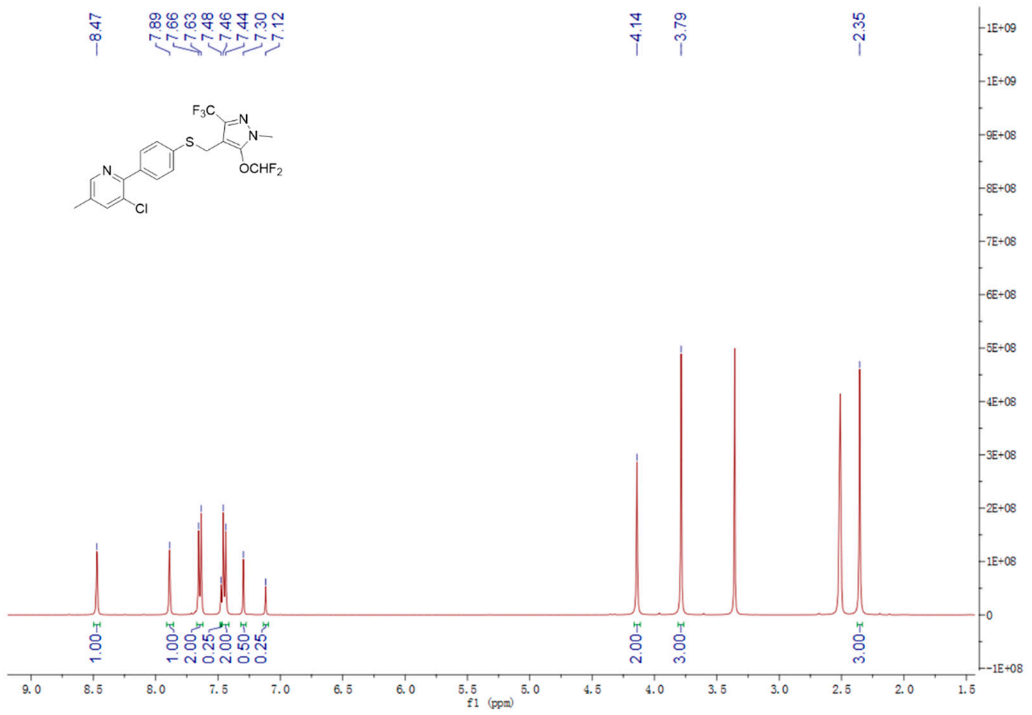

Figure S13. ^{1}H NMR of compound **6e.**

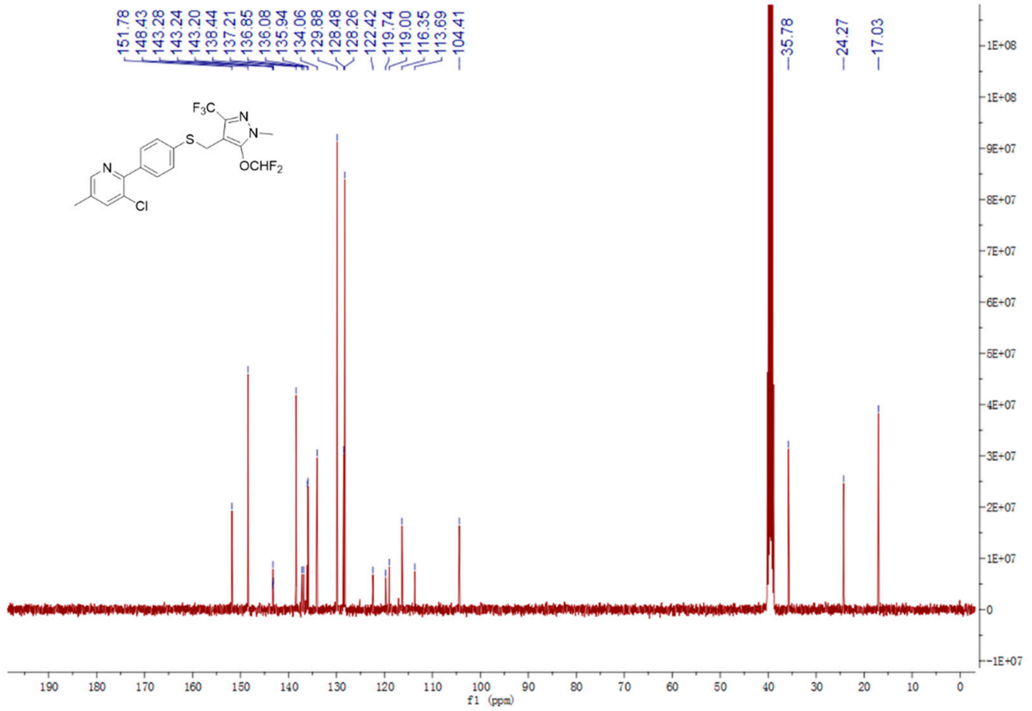

Figure S14. ^{13}C NMR of compound **6e.**

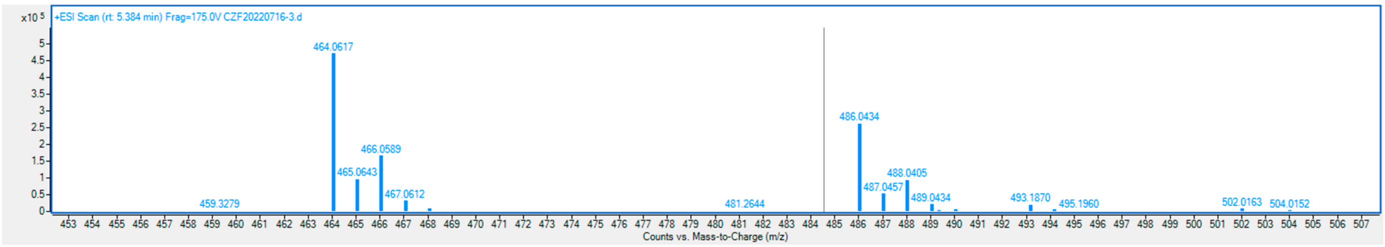

Figure S15. HRMS of compound **6e.**

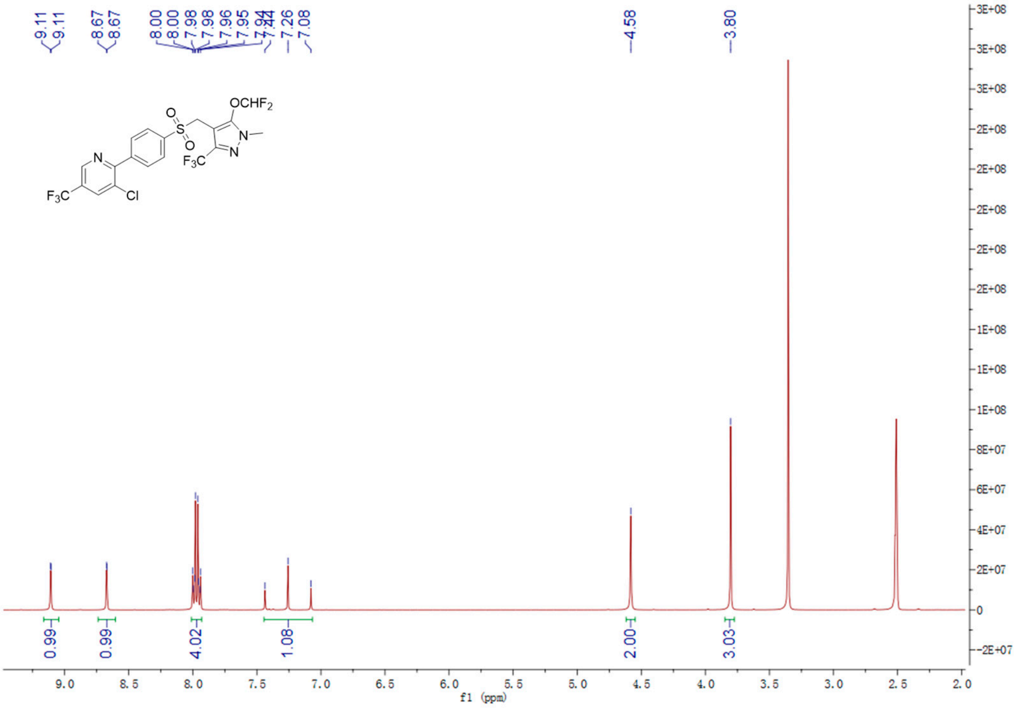

Figure S16. ^{1}H NMR of compound **7a.**

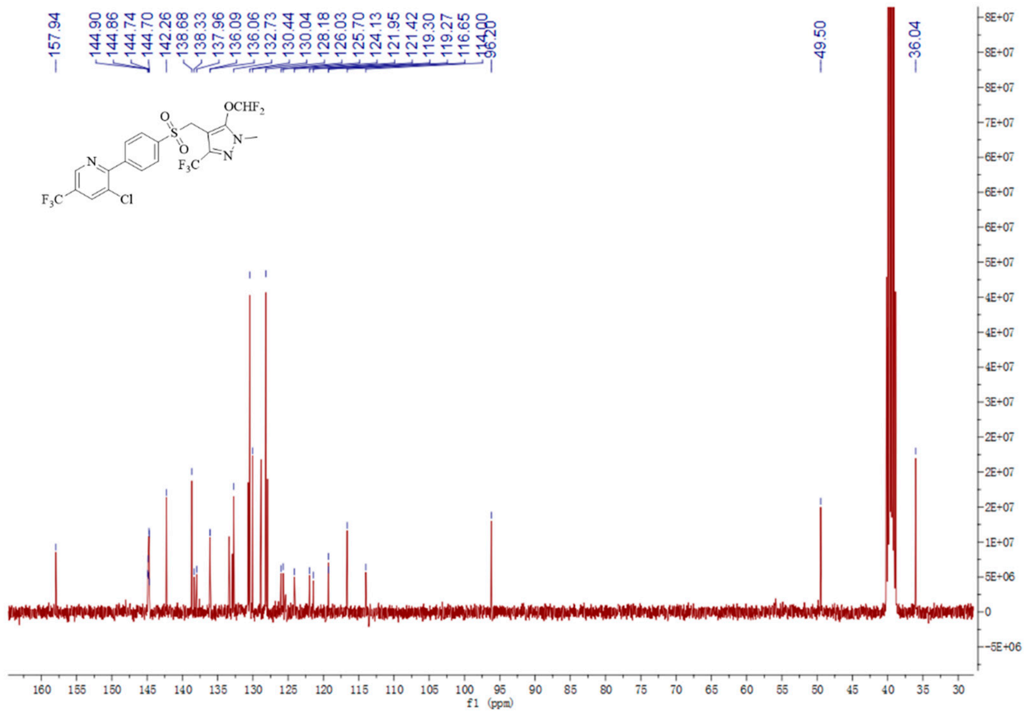

Figure S17. ^{13}C NMR of compound **7a.**

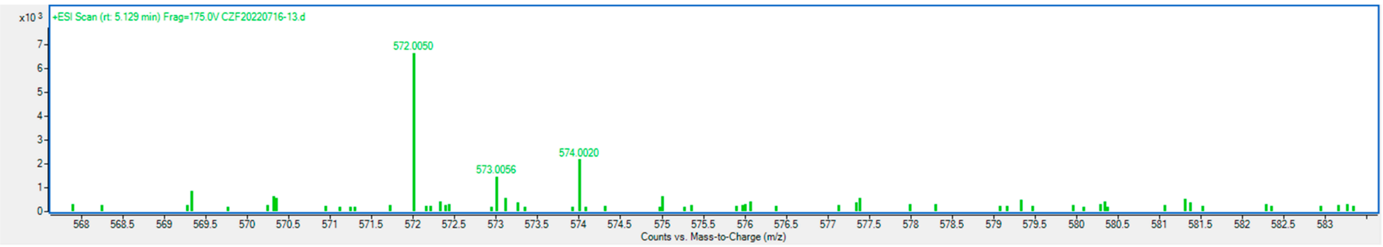

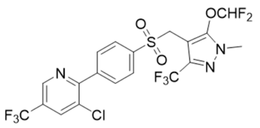

Figure S18. HRMS of compound **7a.**

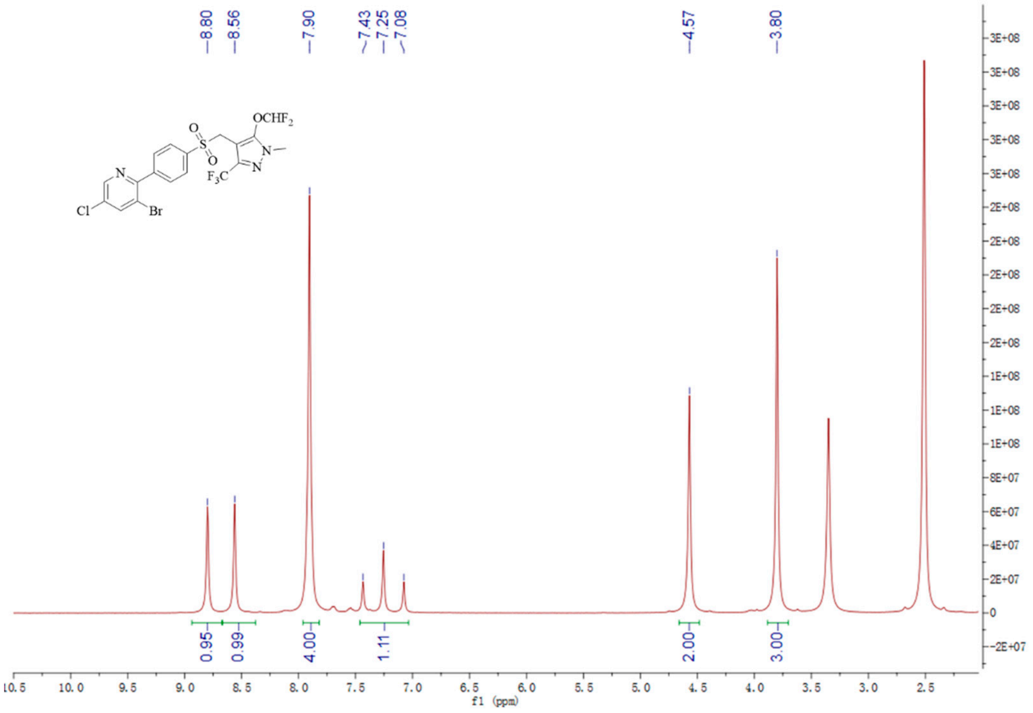

Figure S19. ^{1}H NMR of compound **7b.**

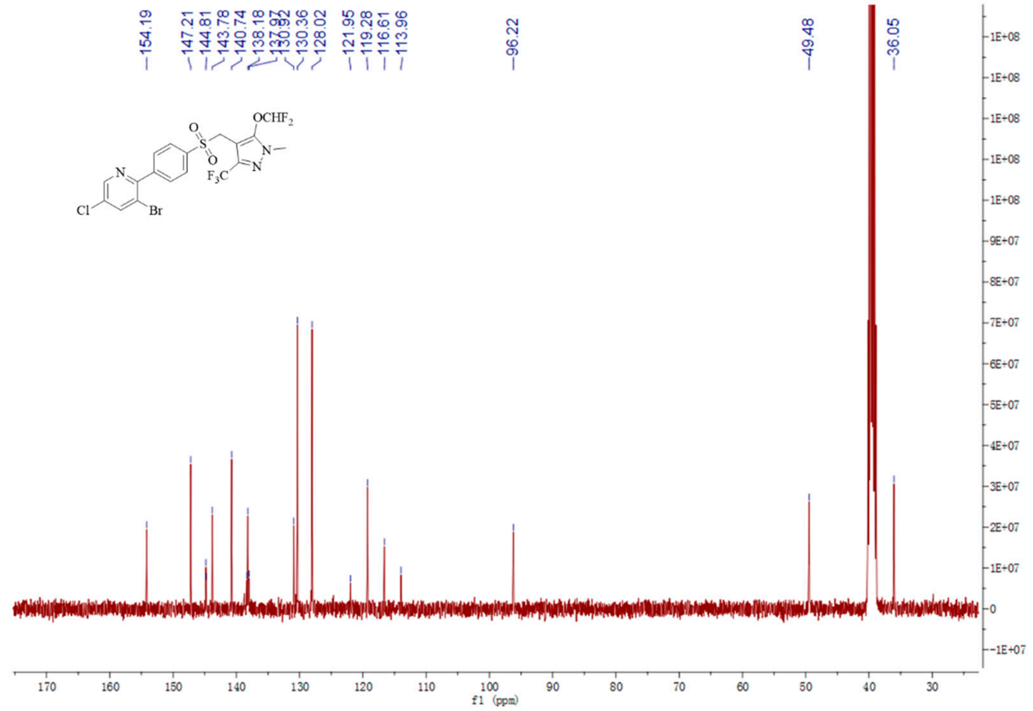

Figure S20. ^{13}C NMR of compound **7b.**

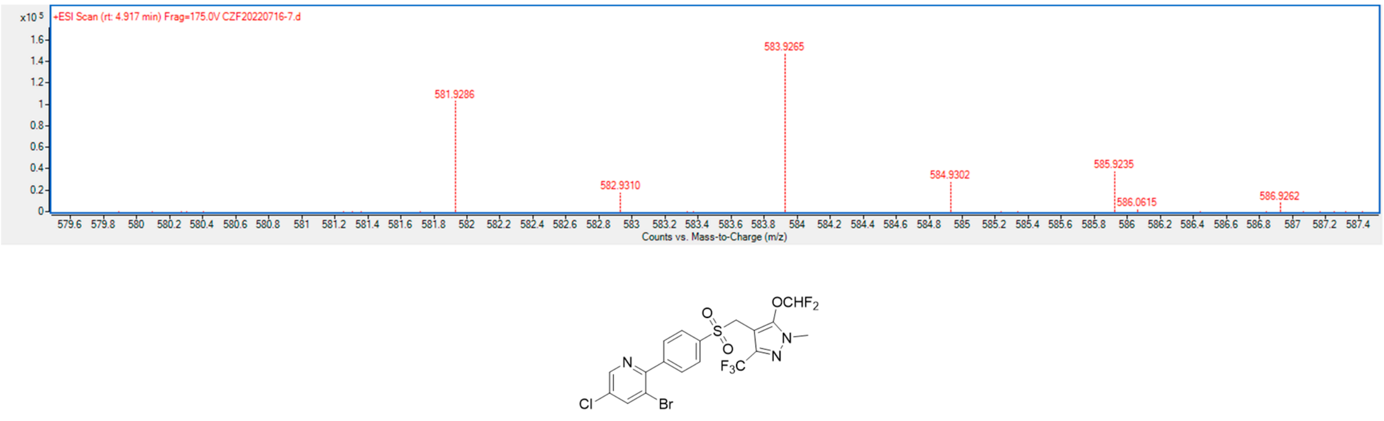

Figure S21. HRMS of compound **7b.**

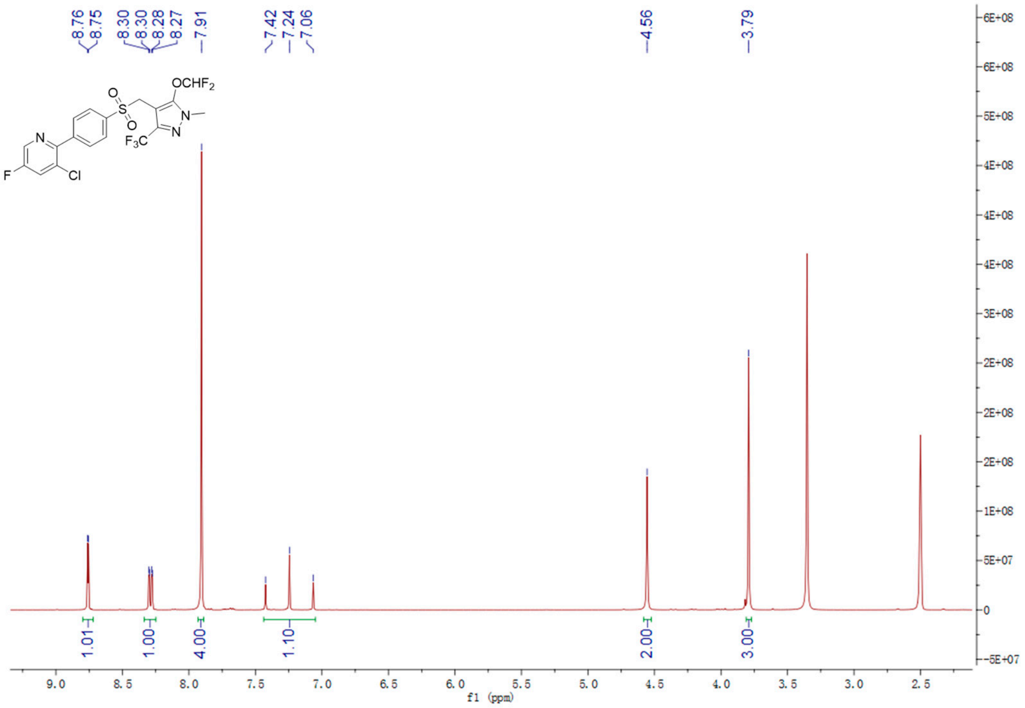

Figure S22. ¹H NMR of compound **7c.**

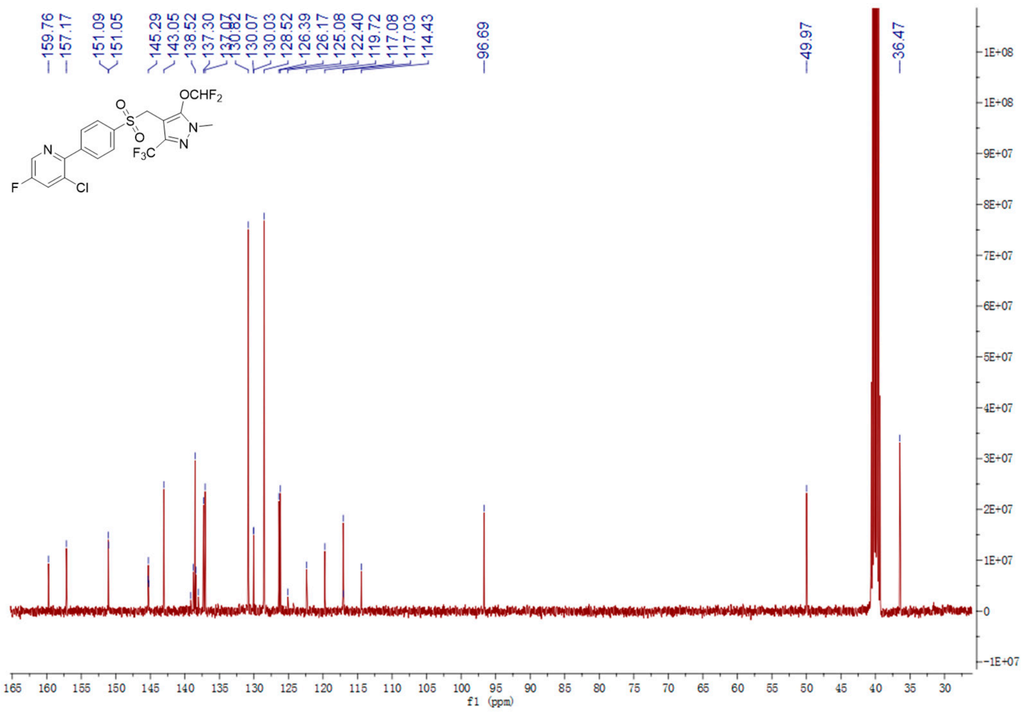

Figure S23. ^{13}C NMR of compound **7c.**

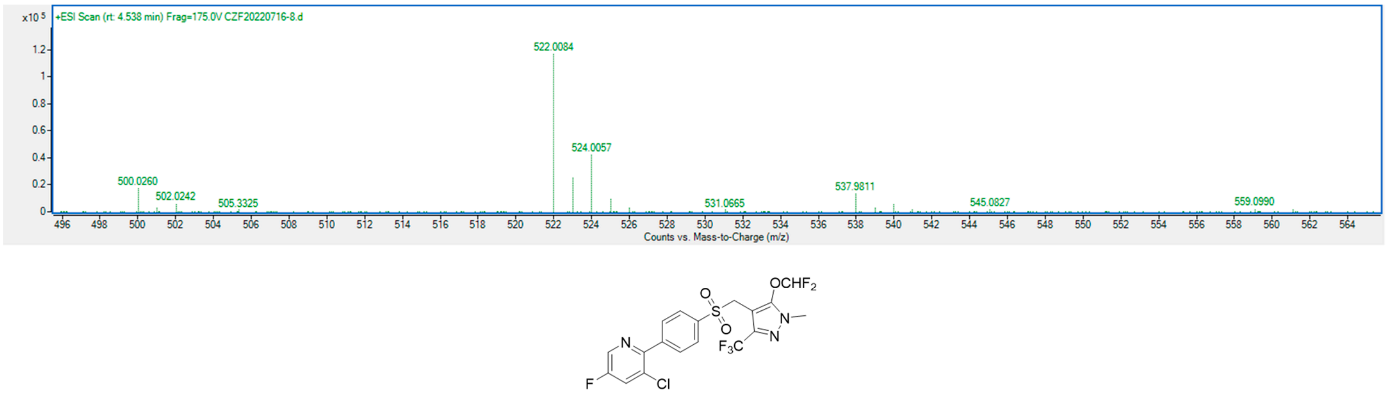

Figure S24. HRMS of compound **7c.**

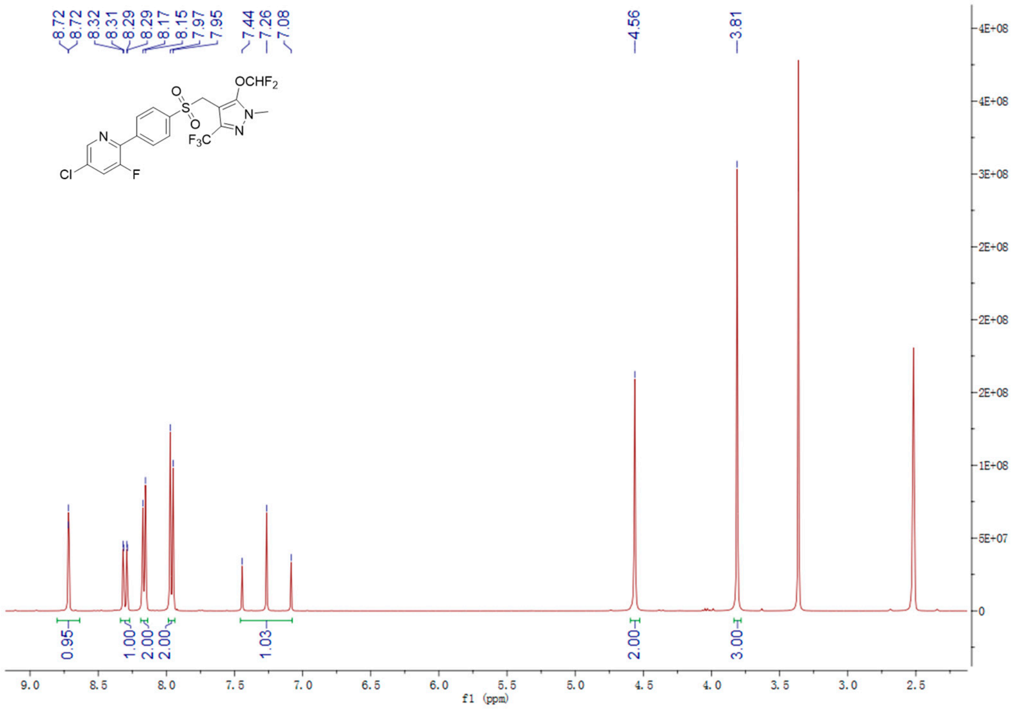

Figure S25. ^{1}H NMR of compound **7d.**

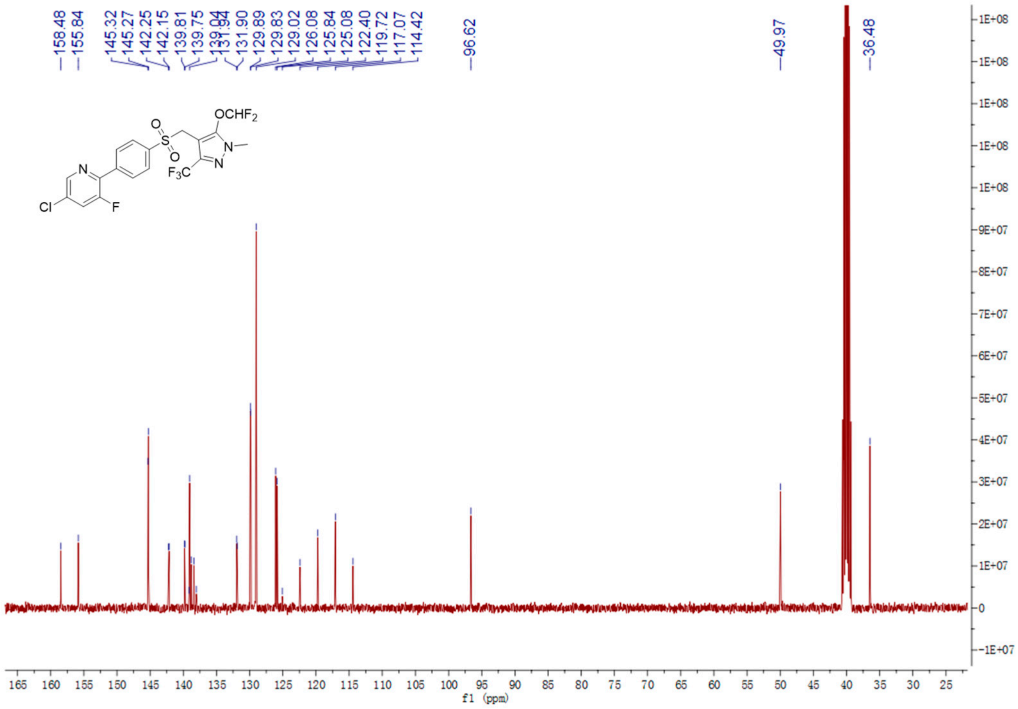

Figure S26. ^{13}C NMR of compound **7d.**

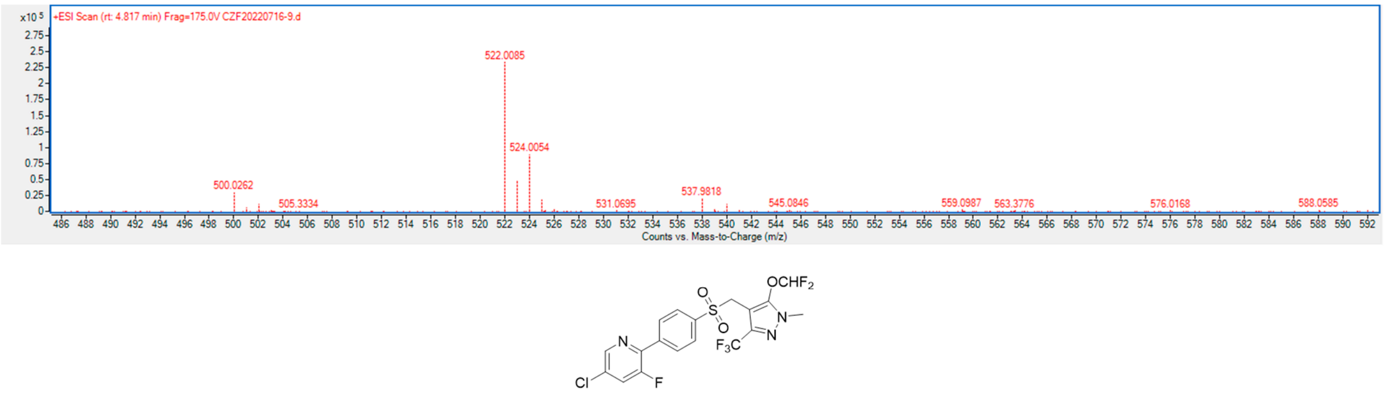

Figure S27. HRMS of compound **7d.**

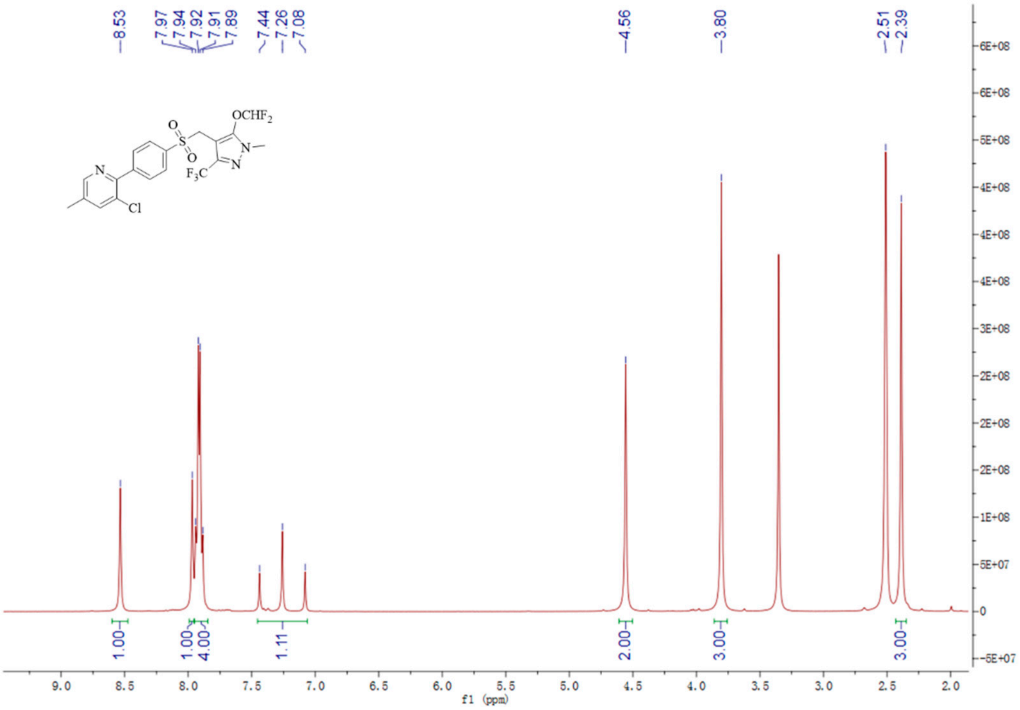

Figure S28. ¹H NMR of compound **7e.**

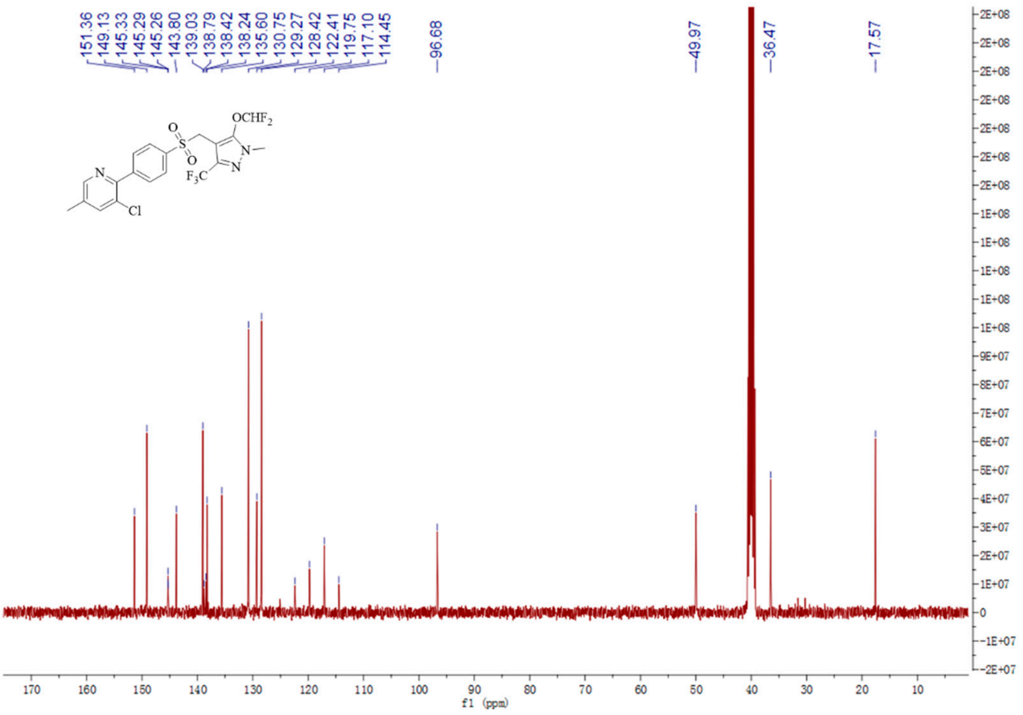

Figure S29. ^{13}C NMR of compound **7e.**

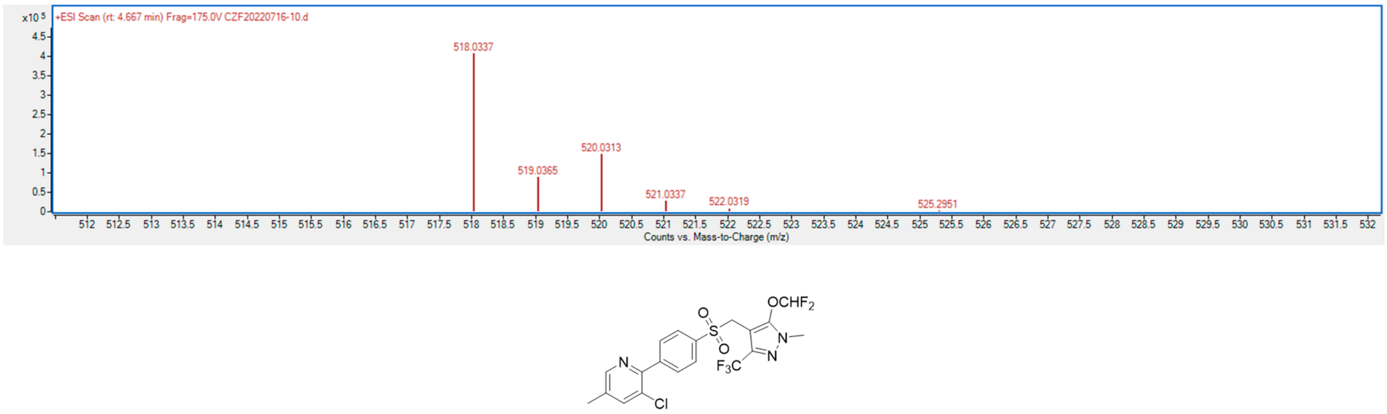

Figure S30. HRMS of compound **7e.**
